# Glycaemic Control in Women With Type 1 Diabetes and Preeclampsia Risk: A Nationwide Cohort Study

**DOI:** 10.1111/1471-0528.18339

**Published:** 2025-08-26

**Authors:** Natalie Holowko, Nathalie Roos, Björn Pasternak, Jonas Söderling, Martin Neovius, Soffia Gudbjörnsdottir, Anna Sandström, Jonas F. Ludvigsson, Olof Stephansson

**Affiliations:** ^1^ Clinical Epidemiology Division, Department of Medicine, Solna Karolinska Institutet Stockholm Sweden; ^2^ Division of Obstetrics and Gynaecology Karolinska University Hospital Stockholm Sweden; ^3^ The Ritchie Centre Hudson Institute of Medical Research Clayton Victoria Australia; ^4^ Department of Epidemiology Research Statens Serum Institut Copenhagen Denmark; ^5^ Department of Medical Epidemiology and Biostatistics Karolinska Institutet Stockholm Sweden; ^6^ Department of Public Health Sciences, Thompson School of Social Work & Public Health University of Hawai'i at Mānoa Honolulu HI USA; ^7^ Department of Molecular and Clinical Medicine, Sahlgrenska Academy, Institute of Medicine University of Gothenburg Gothenburg Sweden; ^8^ Department of Paediatrics Örebro University Hospital Örebro Sweden

**Keywords:** diabetes mellitus, hypertension, preeclampsia, pregnancy, pregnancy complications, pregnancy‐induced, type 1

## Abstract

**Objective:**

No large‐scale studies exist investigating glycaemic control around conception and preeclampsia risk.

**Design:**

Population‐based nationwide cohort study using the National Diabetes Register and other health registers.

**Setting:**

Sweden.

**Population:**

Singleton pregnancies between 2003 and 2019 (*N* = 1 689 301); 4429 with type 1 diabetes (T1DM) and 1 684 872 without.

**Methods:**

The main exposure was having a pre‐gestational diagnosis of T1DM (first diagnosis anytime ≤ 91 days after conception and ≥ 1value of glycated haemoglobin (HbA1c) within ±90 days from conception). Peri‐conceptional glycaemic control was categorised using the most recent HbA1c measurement (mmol/mol: < 48; 48–61; 62–75; ≥ 76). Unexposed were women without any diabetes diagnosis (≤ 91 days after conception).

**Main Outcome Measures:**

Preeclampsia was defined using ICD codes and further categorised according to completed gestational weeks at delivery with preeclampsia diagnosis: early preterm preeclampsia (< 34 + 0), late preterm preeclampsia (34 + 0 to 36 + 6), or term preeclampsia (≥ 37 + 0).

**Results:**

16.8% of women with T1DM developed preeclampsia, compared to 2.9% of women without diabetes (adjusted RR [aRR] 4.7, 95% CI 4.4–5.0). Preeclampsia risk increased with peri‐conceptional HbA1c, from 11.6% in women with HbA1c < 48 mmol/mol to 23.3% in women with HbA1c ≥ 76 mmol/mol. Compared to unexposed women, there was a dose–response relationship between HbA1c and preeclampsia in women with T1DM (HbA1c < 48 mmol/mol aRR 3.4 (2.9–4.0); HbA1c 48–61 mmol/mol aRR 4.6 (4.2–5.1); HbA1c 62–75 mmol/mol aRR 5.7 (5.0–6.5): HbA1c ≥ 76 mmol/mol aRR 6.3 (CI 5.3–7.7)). Compared to unexposed women, the aRR of term preeclampsia was 3.5 times higher (3.1–3.9) in women with T1DM, while the aRR was much higher for early preterm preeclampsia (aRR 7.2: 6.1–8.5) and late preterm preeclampsia (aRR 9.9: 8.8–11.1).

**Conclusions:**

Women with T1DM had a higher risk of preeclampsia, which increased in a dose–response manner with poorer peri‐conceptional glycaemic control.

**Abbreviations**
HbA1c: glycated haemoglobin
T1DM: Type 1 diabetes mellitus

## 1 Introduction

While Type 1 Diabetes Mellitus (T1DM) can be diagnosed at any age, it is typically diagnosed during childhood and adolescence and requires lifelong insulin treatment. The global incidence of T1DM has increased in recent decades [1, 2], particularly in the Nordic countries [3, 4]. A Swedish population‐based study found that 0.5% of pregnancies from 1998 to 2012 occurred in mothers with pre‐gestational T1DM [5], with a 33.2% increase in T1DM prevalence in women who gave birth during this 15‐year period [5]. Poor glucose control around conception in women with pre‐pregnancy diabetes has been linked to adverse maternal and perinatal outcomes [6, 7, 8]. Children of mothers with T1DM have an increased risk of congenital malformations [6, 9], preterm birth [7], stillbirth and neonatal death [10, 11]. Additionally, T1DM is associated with higher risks of gestational hypertension and preeclampsia [12, 13, 14, 15].

Preeclampsia is a systemic vascular disorder and a leading cause of maternal and perinatal mortality and morbidity globally [16, 17], currently without any treatment except delivery of the foetus and placenta. Preeclampsia is characterised by hypertension accompanied by proteinuria or other organ dysfunction during the second half of pregnancy, affecting the kidneys, liver, haematological system, or brain [18]. Preeclampsia causes placental insufficiency, which can lead to foetal growth restriction [18]. Strong evidence shows that preeclampsia morbidity extends beyond pregnancy, increasing long‐term maternal health risks, including cardiovascular and cerebrovascular diseases and death [19, 20]. Therefore, prophylactic measures and optimisation of modifiable risk factors play a crucial role.

The health impact of T1DM during pregnancy is variable. Endothelial dysfunction can lead to cardiovascular issues, including hypertension and kidney complications, like microalbuminuria or diabetic nephropathy [21]. While some smaller studies report adverse maternal and neonatal outcomes in women with T1DM, even when glycaemic control at the start of pregnancy is optimal, there is limited evidence on the effect of pre‐pregnancy glycaemic control on preeclampsia risk [13, 22, 23, 24]. Improved glucose control is likely to decrease the risk of preeclampsia and represent an important target for intervention. One small study (120 women with T1DM in one region of Sweden) used continuous glucose monitoring and found no association between glycaemic control during pregnancy and the risk of preeclampsia [25]. Although HbA1c is a strong marker of glycaemic control, no large‐scale studies to date have investigated glycaemic control around conception and preeclampsia risk [24, 26, 27, 28].

Using a nationwide cohort, we investigated the association between levels of glycated haemoglobin (HbA1c) around conception among women with T1DM and the risk of preeclampsia.

## 2 Materials and Methods

### 2.1 Study Design and Participants

This population‐based cohort study used Swedish nationwide health registers with prospectively collected data: Medical Birth Register (MBR) [29], National Diabetes Register (NDR) [30], National Patient Register [31], Cause of Death Register [32], Total Population Register [33], The Longitudinal Integrated Database for Health Insurance and Labour Market Studies (LISA) [34] and Prescribed Drug Register [35]. Data were linked using the unique personal identity number assigned to each individual in Sweden at birth or immigration [36].

**FIGURE 1:**
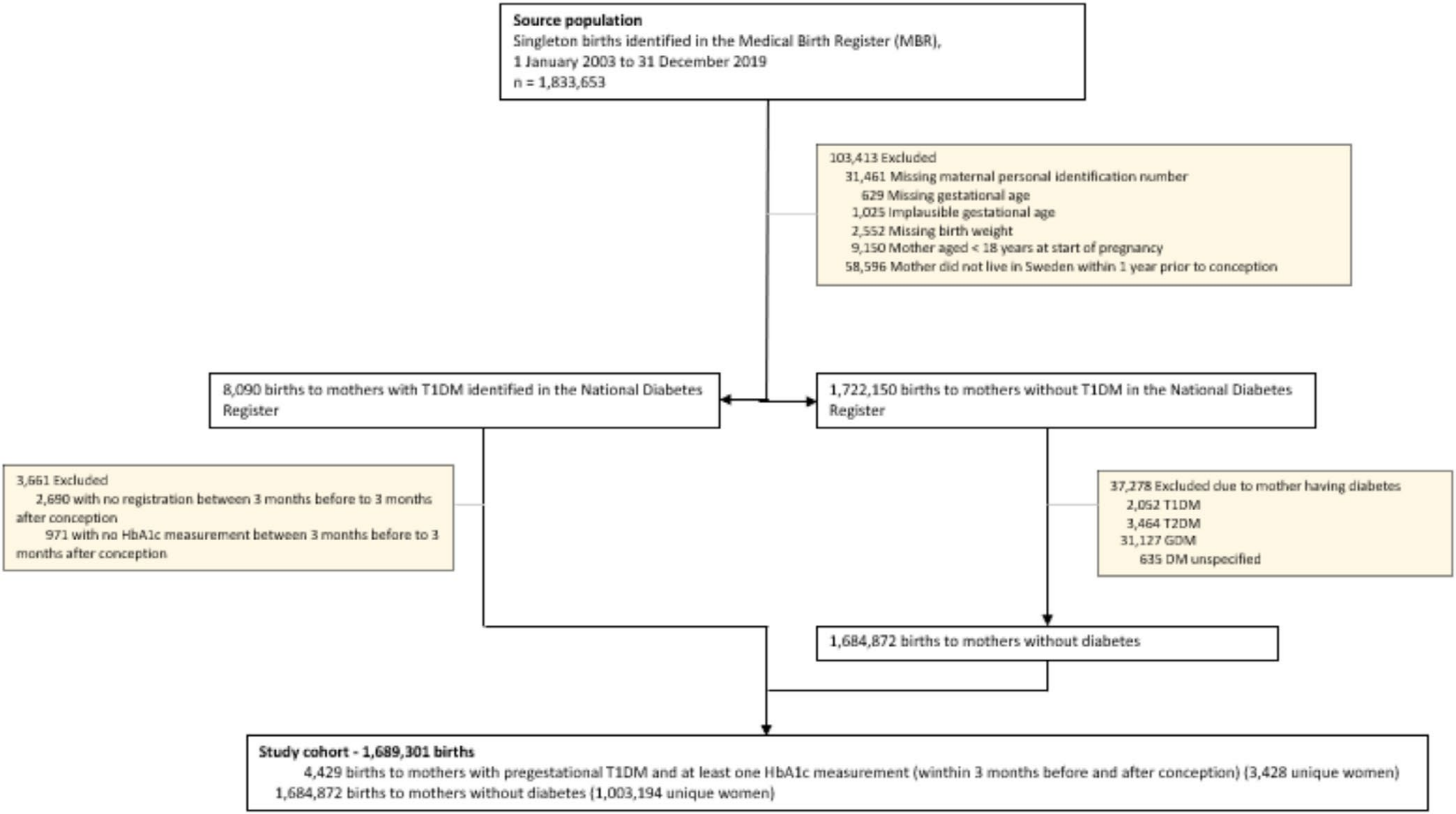
Flowchart of cohort selection. DM, diabetes mellitus; GDM, gestational diabetes mellitus; HbA1c, haemoglobin A1c (glycated haemoglobin); T1DM, Type 1 diabetes mellitus; T2DM, Type 2 diabetes mellitus.

Our study population was drawn from all singleton births (live and stillbirths) in Sweden between 1 January 2003 and 31 December 2019 (*N* = 1 833 653), identified in the MBR. We then excluded women: missing a personal identity number (allowing linkages); missing gestational age at birth or with an implausible gestational age (≤ 22 + 0 and > 44 + 0 gestational weeks); age < 18 years at conception; and non‐residents in Sweden in the year preceding conception. Our study cohort consisted of 1 689 301 singleton births (Figure 1), which included 1 684 872 births to women without T1DM or any other type of diabetes (99.73% of the final sample) and 4429 births (0.26%) to women with T1DM who had a peri‐conceptional HbA1c measurement.

The first day of the last menstrual period was used as an estimate of conception, calculated as the difference between the date of birth and gestational age at birth. Gestational age was captured from the MBR using a hierarchical definition using: (1) ultrasonography, (2) date of last menstrual period, and (3) clinical estimation at birth. Early second‐trimester ultrasonography for determining gestational age has been used routinely in Sweden since 1990 and is performed in more than 95% of pregnancies [37].

### 2.2 Exposure

HbA1c is a strong marker of glycaemic control and we used pre‐conceptional data as a proxy for glycaemic control during pregnancy. Using the NDR, women with a diagnosis of T1DM anytime up to 91 days after conceiving the index pregnancy who also had at least one HbA1c value (within 3 months before and after conception) were identified as having pre‐gestational T1DM (Table S1). For analyses of glycaemic control in women with T1DM, the most recent HbA1c measurement was categorised in mmol/mol (< 48; 48–61; 62–75; or ≥ 76). The lowest HbA1c level (< 48 mmol/mol represents the ‘target level’ at pre‐conception recommended in Sweden) [38], while the remaining categories arbitrarily represent moderate to high risk. Unexposed women were defined as women with a singleton pregnancy who did not have T1DM nor any other type of diabetes up to 91 days after conception.

In addition to investigating the absolute and relative risk of preeclampsia among women with T1DM by HbA1c categories, we also used alternative measures of poor glycaemic control or diabetes severity for women with T1DM (Table S2).

### 2.3 Outcomes

Our main outcome of preeclampsia was identified using clinical diagnoses (made by an obstetrician) in the MBR and/or National Patient Register, using the Swedish version of the International Classification of Diseases, 10th revision (ICD‐10) (Table S1). The ICD‐10 codes O11 and O14‐O15 were used to identify preeclampsia as: hypertension (two instances of blood pressure ≥ 140 mmHg and/or diastolic blood pressure ≥ 90 mmHg with an interval of at least 4 h), combined with proteinuria (≥ 0.3 g/24 h) occurring at ≥ 20 + 0 weeks gestation; else as superimposed preeclampsia (chronic hypertension with the addition of proteinuria) or eclampsia. In contrast to the contemporary definition [39], proteinuria was mandatory (alongside hypertension) in order to meet a diagnosis of preeclampsia during the study period. We defined women as having preeclampsia if they had any of the aforementioned ICD diagnoses: ≥ 1 diagnosis in the inpatient register or ≥ 2 diagnoses in the outpatient register (between gestational Week 20 to 1 week postpartum); or 1 diagnosis in the outpatient register and 1 diagnosis in the MBR. Preeclampsia identified in the patient register has high validity (92.6% positive predictive value) [31].

In terms of severity and gestation, the heterogeneous manifestation of preeclampsia can lead to severe illness in the mother and foetal growth restriction, both of which would indicate the need for early delivery [40]. Given this, we stratified women with a preeclampsia diagnosis according to gestational age at delivery (completed gestational weeks)—early preterm preeclampsia (< 34 + 0), late preterm preeclampsia (34 + 0 to 36 + 6), or term preeclampsia (≥ 37 + 0).

### 2.4 Covariates

We retrieved the following information from the MBR: Maternal age at conception (years) presented categorically (18–24, 25–29, 30–34, 35–39, ≥ 40), modelled as a continuous variable; Cohabitation status (not cohabiting vs. cohabiting); Country of birth (‘Nordic’ or ‘Other’); Year of birth (2003–2009, 2010–2014 or 2015–2019); Highest achieved education (≤ 9, 10–12, ≥ 13 years); Parity (nulliparous, 1, ≥ 2 previous births); WHO body mass index (BMI) classification (< 18.5, 18.5–< 25, 25–< 30, 30–< 35, ≥ 35 kg/m^2^) [41]; Smoking in early pregnancy (non‐smoker, 1–9 cigarettes or ≥ 10 cigarettes per day); Assisted reproductive technology (ART‐dichotomous), including in vitro fertilisation (IVF) and intracytoplasmic sperm injection (ICSI), but no other form of assisted reproduction (such as ovulation induction or insemination). Pre‐pregnancy hypertension was identified in the National Patient Register and/or the MBR using the ICD‐10 codes O10, I10‐15, or the ATC codes C02, C03, C07, C08, C09 from the Prescribed Drug Register. Information on co‐existing autoimmune diseases was identified in the National Patient Register (Table S3).

### 2.5 Statistical Analyses

Maternal characteristics of women with T1DM and women without diabetes were described using frequencies and percentages for categorical variables and means and standard deviations (SDs) for continuous variables, stratified by levels of HbA1c for women with T1DM. We used generalised estimating equations assuming a Poisson distribution with a log link and an exchangeable covariance matrix, accounting for multiple unique pregnancies per woman (repeated statement) and resulting in robust estimates of the standard error [42]. We estimated crude and adjusted risk ratios (RR) of preeclampsia by HbA1c levels in women with T1DM versus those without diabetes, as well as by parity. Absolute risk difference (percentage points) was calculated as ([adjusted]OR‐1) × the proportion in the comparator group. Multivariable models adjusted for birth year, maternal age, BMI, parity, living with a partner, smoking, country of birth, education level, ART, and diabetes‐related autoimmune diseases (Table S3). We used the Prescribed Drug Register to identify women who (from 2006 onwards, due to data availability) used aspirin during their pregnancy (Table S3) and used ICD‐10 codes to identify women who had a record of albuminuria or ketoacidosis (Table S2), specifically, in the last 12 months before conception. Mode imputation was used for the 0.6% of women with missing education (imputed as ≥ 13 years). Missing values were coded as ‘No’ for living with a partner (missing 4.9%) and ‘Other’ for country of birth (0.1%). For BMI and smoking, we used multiple imputation with the fully conditional specification method (five iterations) [43]. Variables in the imputation model included calendar year of birth, maternal age, country of birth, living with a partner, education, parity, BMI, maternal height, smoking status and diabetes‐related autoimmune diseases. We also described our population using non‐imputed data (Table S4).

We also examined preeclampsia risk in women with T1DM using alternative markers of poor glycaemic control and diabetes severity, including (1) hospital admission for acidosis or T1DM in the year before conception, (2) long‐term glycaemic control (average of last 3 HbA1c values, most retrieved earlier than 90 days before conception), and (3) markers of renal function: albuminuria and estimated glomerular filtration rate (eGFR; < 60 mL/min/1.73 m^2^, 60–120 mL/min/1.73 m^2^, and > 120 mL/min/1.73 m^2^).

We also compared excluded women with T1DM who lacked HbA1c data around conception to those in the study population with HbA1c values (Table S5). We ran additional sensitivity analyses adjusting for aspirin use during pregnancy (for births in 2006 onwards, due to data availability) and also excluding women with a record of albuminuria in the last 12 months before conception. Estimates are presented with 95% confidence intervals (CI). Analyses were conducted using SAS (version 9.4) and STATA (version 17.0).

## 3 Results

Among 1 684 872 births to 1 003 194 unique women without diabetes and 4429 births to 3428 unique women with T1DM, the mean age at delivery was 30.2 years and 29.9 years, respectively (Table 1, and same in our non‐imputed sample Table S4). Women with T1DM were more likely to be Nordic born than those without T1DM (94.1% vs. 79.5%). Living without a partner was slightly less common in women with T1DM (9.6% vs. 10.5%). Among women with T1DM, the proportion with a high education decreased gradually with increasing HbA1c levels. Having a BMI ≥ 25 was more prevalent in women with T1DM than those without (56.5% vs. 41.6%) and rose with higher HbA1c levels. Among women with T1DM, smoking was much more common in women with HbA1c ≥ 76 mmol/mol, compared to those with HbA1c < 48 mmol/mol (21.0% vs. 3.0%). Compared to women with T1DM, a smaller proportion of women with T1DM who were excluded (due to no HbA1c value) were Nordic born (91% vs. 94%) or nulliparous (44% vs. 49%) (Table S5).

**TABLE 1:** Maternal characteristics stratified by type1 diabetes mellitus (T1DM) status and levels of glycated haemoglobin (HbA1c) (*N* = 1 689 301).

Characteristics	Mothers without diabetes (*n* = 1 684 872^a^)	Mothers with T1DM, stratified by levels of glycated haemoglobin (HbA1c)				
		Total (*n* = 4429^b^)	HbA1c < 48 mmol/mol (*n* = 1118)	HbA1c 48–61 mmol/mol (*n* = 2037)	HbA1c 62–75 mmol/mol (*n* = 892)	HbA1c ≥ 76 mmol/mol (*n* = 382)
Age, years, mean (SD)	30.2 (5.0)	29.9 (4.8)	30.1 (4.4)	30.3 (4.7)	29.6 (5.1)	27.8 (5.3)
18–24.9	272 719 (16.2)	697 (15.7)	126 (11.3)	262 (12.9)	181 (20.3)	128 (33.5)
25–29	554 415 (32.9)	1620 (36.6)	431 (38.6)	747 (36.7)	310 (34.8)	132 (34.6)
30–34	561 320 (33.3)	1409 (31.8)	396 (35.4)	678 (33.3)	256 (28.7)	79 (20.7)
35–39	251 954 (15.0)	603 (13.6)	143 (12.8)	302 (14.8)	122 (13.7)	36 (9.4)
≥ 40	44 464 (2.6)	100 (2.3)	22 (2.0)	48 (2.4)	23 (2.6)	7 (1.8)
Country of birth						
Nordic	1 339 080 (79.5)	4167 (94.1)	1051 (94.0)	1936 (95.0)	832 (93.3)	348 (91.1)
Other/missing	345 792 (20.5)	262 (5.9)	67 (6.0)	101 (5.0)	60 (6.7)	34 (8.9)
Living with a partner						
Yes	1 507 885 (89.5)	4003 (90.4)	1037 (92.8)	1872 (91.9)	786 (88.1)	308 (80.6)
No/missing	176 987 (10.5)	426 (9.6)	81 (7.2)	165 (8.1)	106 (11.9)	74 (19.4)
Year of birth						
2003–2009	658 494 (39.1)	1116 (25.2)	262 (23.4)	500 (24.5)	254 (28.5)	100 (26.2)
2010–2014	510 144 (30.3)	1378 (31.1)	293 (26.2)	624 (30.6)	306 (34.3)	155 (40.6)
2015–2019	516 234 (30.6)	1935 (43.7)	563 (50.4)	913 (44.8)	332 (37.2)	127 (33.2)
Level of education, years						
≤ 9	173 062 (10.3)	358 (8.1)	50 (4.5)	127 (6.2)	97 (10.9)	84 (22.0)
10–12	646 359 (38.4)	1810 (40.9)	372 (33.3)	791 (38.8)	436 (48.9)	211 (55.2)
≥ 13	865 451 (51.4)	2261 (51.0)	696 (62.3)	1119 (54.9)	359 (40.2)	87 (22.8)
Parity						
0	731 826 (43.4)	2157 (48.7)	567 (50.7)	1008 (49.5)	406 (45.5)	176 (46.1)
1	629 382 (37.4)	1629 (36.8)	432 (38.6)	746 (36.6)	318 (35.7)	133 (34.8)
≥ 2	323 664 (19.2)	643 (14.5)	119 (10.6)	283 (13.9)	168 (18.8)	73 (19.1)
Body mass index,^c^ kg/m^2^						
< 18.5	47 668 (2.8)	41 (0.9)	8 (0.7)	17 (0.8)	10 (1.2)	6 (1.6)
18.5–< 25	989 174 (58.7)	2043 (46.1)	577 (51.6)	926 (45.4)	384 (43.1)	156 (40.8)
25–< 30	439 594 (26.1)	1598 (36.1)	388 (34.7)	748 (36.7)	325 (36.4)	138 (36.0)
30–< 35	150 708 (8.9)	564 (12.7)	114 (10.2)	268 (13.2)	122 (13.6)	60 (15.7)
≥ 35	57 727 (3.4)	183 (4.1)	30 (2.7)	79 (3.9)	51 (5.7)	23 (5.9)
ART^d^	54 393 (3.2)	160 (3.6)	57 (5.1)	75 (3.7)	25 (2.8)	3 (0.8)
Smoking in early pregnancy						
Smoker	110 710 (6.6)	275 (6.2)	34 (3.0)	80 (3.9)	81 (9.1)	80 (21.0)
Non‐smoker	1 574 162 (93.4)	4154 (93.8)	1084 (97.0)	1957 (96.1)	811 (90.9)	302 (79.0)
Pre‐pregnancy hypertension	66 688 (4.0)	812 (18.3)	145 (13.0)	378 (18.6)	192 (21.5)	97 (25.4)
Other diabetes‐related autoimmune diseases	35 464 (2.1)	595 (13.4)	144 (12.9)	256 (12.6)	140 (15.7)	55 (14.4)
N deliveries from 2006	1 412 826 (83.9)	4075 (92.0)	1038 (92.8)	1881 (92.3)	809 (90.7)	347 (90.8)
Aspirin use during pregnancy	22 699 (1.6)	374 (9.2)	103 (9.9)	167 (8.9)	76 (9.4)	28 (8.1)
Albuminuria^f^ within 12 months before conception	132 (0.0)	788 (17.8)	141 (12.6)	342 (16.8)	201 (22.5)	104 (27.2)
Hypoglycaemia within 12 months before conception	59 (0.0)	72 (1.6)	13 (1.2)	33 (1.6)	20 (2.2)	6 (1.6)
Ketoacidosis^e^ within 12 months before conception	—	78 (1.8)	10 (0.9)	27 (1.3)	17 (1.9)	24 (6.3)
Median duration T1DM (IQR), years	—	16 (9–22)	14 (5–20)	16 (10–22)	16 (11–22)	15 (10–20)
Median HbA1c level (IQR), mmol/mol	—	55 (47–64)	43 (39–45)	54 (51–58)	67 (64–70)	83 (79–91)

Abbreviations: HbA1c, haemoglobin A1c (glycated haemoglobin); IQR, interquartile range; SD, standard deviation; T1DM, Type 1 diabetes mellitus.

^a^ 1 003 194 unique women without diabetes.

^b^ 3428 unique women with diabetes.

^c^ Body mass index in early pregnancy.

^d^ Assisted reproductive technology.

^e^ Ketoacidosis defined using ICD‐10 codes (E10.0A, E10.1).

^f^ Albuminuriadefined as Normoalbuminuria, Mircoalbuminuria, or Macroalbuminuria based on the last value in the National Diabetes Register‐Microalbuminuria (≥ 2 positive results from 3 urine samples obtained within 1 year, withpositivity defined as an albumin‐to‐creatinine ratio of 3–30 mg/mmol (roughly 30–300 mg/g) or urinary albumin clearance of 20–200 μg/min (20–300 mg/L)); Macroalbuminuria (having an albumin‐to‐creatinine ratio of > 30 mg/mmol (roughly ≥ 300 mg/g) or urinary albumin clearance of > 200 μg/min (> 300 mg/L)).

### 3.1 HbA1c Levels and Preeclampsia Risk

**FIGURE 2:**
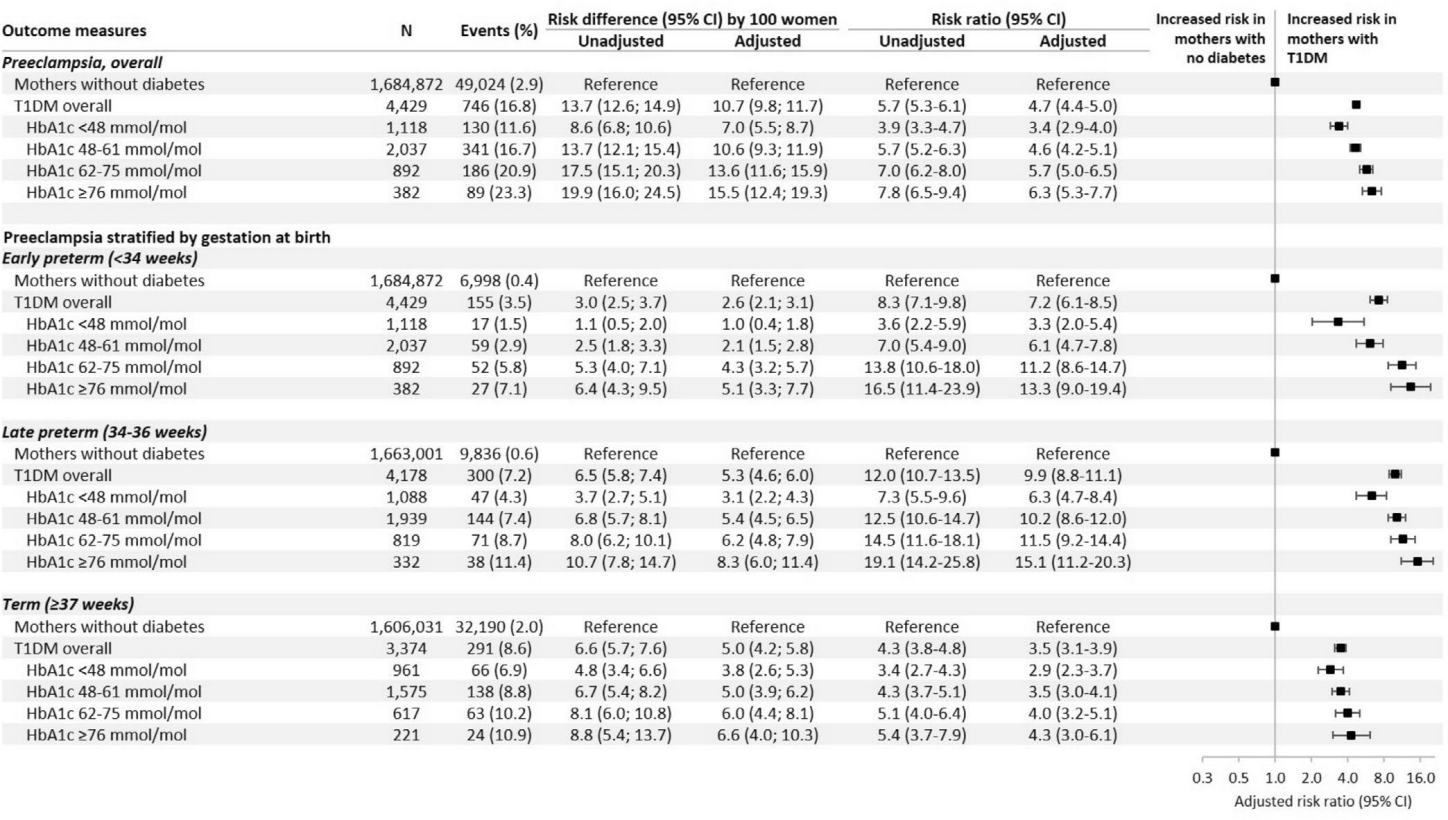
Risk ratios and risk differences of preeclampsia by Type 1 diabetes mellitus (T1DM) status and levels of glycated haemoglobin (HbA1c), overall and stratified by gestation at birth for women with preeclampsia. CI, confidence interval; HbA1c, haemoglobin A1c (glycated haemoglobin). *Adjusted for year of birth, maternal age, country of birth, living with a partner, highest achieved education, parity, body mass index, assisted reproductive technology, smoking status and other diabetes‐related autoimmune diseases.

Of the 4429 women with T1DM, 16.8% developed preeclampsia, compared to 2.9% of the 1 684 872 women without diabetes (Figure 2). The absolute risk of preeclampsia increased with increasing HbA1c levels, from 11.6% for HbA1c < 48 mmol/mol to 23.3% for HbA1c ≥ 76 mmol/mol. This absolute risk was much larger for nulliparous than parous women (17.3% vs. 5.8% for HbA1c < 48 mmol/mol and 33.5% vs. 14.6% for HbA1c ≥ 76 mmol/mol) (Figures S1 and S2).

The adjusted risk ratio (aRR) of developing preeclampsia was 4.7 times higher (95% CI 4.4–5.0) in women with T1DM, compared to those without (Figure 2). The aRR increased with higher HbA1c levels, from 3.4 (2.9–4.0) for HbA1c levels < 48 mmol/mol to 6.3 (5.3–7.7) for HbA1c levels ≥ 76 mmol/mol. Likewise, compared to women without diabetes, the adjusted risk difference (aRD) of preeclampsia for women with T1DM increased with increasing HbA1c levels from 7% for HbA1c < 48 mmol/mol to 15.5% for HbA1c ≥ 76 mmol/mol. When stratified by parity, the aRD was larger for nulliparous (Figure S1, aRD 15.7, 14.2–17.3) than for parous women (Figure S2, aRD 7.4, 95% CI 6.3–8.6), with these differences more pronounced for late preterm and term preeclampsia.

In sensitivity analyses (1) adjusting for aspirin use; and (2) excluding women with a record of albuminuria in the last 12 months before conception, we found slightly lower estimates; however, both the direction and interpretation of our main findings remained the same (Figure S5).

### 3.2 HbA1c Levels and Preeclampsia Risk, Stratified by Gestation at Birth

Of the 4429 women with T1DM, 3.5% developed early preterm preeclampsia, compared to 0.4% of the 1 684 872 women without diabetes (Figure 2). The aRR of early preterm preeclampsia was 7.2 times higher (6.1–8.5) in women with T1DM, with the absolute risk increasing from 1.5% at HbA1c < 48 mmol/mol to 7.1% at HbA1c ≥ 76 mmol/mol and the aRR increasing from 3.3 (2.0–5.4) to 13.3 (9.0–19.4).

Of the remaining 4178 women with T1DM, 7.2% developed late preterm preeclampsia (delivery 34–36 gestational weeks), compared to 0.6% of women without diabetes (Figure 2). The aRR of late preterm preeclampsia for women with T1DM was 9.9 (8.8–11.1). This risk increased with higher HbA1c levels, with both incidence and aRR rising from 4.3% (aRR 6.3, 4.7–8.4) for HbA1c < 48 mmol/mol to 11.4% (aRR 15.1, 11.2–20.3) for HbA1c ≥ 76 mmol/mol.

Of the remaining 3374 women with T1DM, 8.6% developed term preeclampsia (delivery ≥ 37 gestational weeks), compared to 2% of women without diabetes (Figure 2). The aRR of term preeclampsia for women with T1DM was 3.5 (3.1–3.9). This risk increased with higher HbA1c levels, with both the incidence and aRR rising from 6.9% (aRR 2.9, 2.3–3.7) for HbA1c < 48 mmol/mol to 10.9% (aRR 4.3, 3.0–6.1) for HbA1c ≥ 76 mmol/mol.

### 3.3 Alternative Measures of Poor Glycaemic Control and Preeclampsia Risk

There was not strong evidence to suggest that women with T1DM admitted to hospital for acidosis (in the year before conception) had a higher risk of preeclampsia (aRR 5.4, 3.2–9.0), compared to those not admitted (aRR 4.7, 4.4–5.0) (Table 2). There was no difference in the risk of preeclampsia in women with T1DM who were admitted to hospital for diabetes (aRR 4.5, 3.4–6.0), compared to those who were not admitted (aRR 4.7, 4.4–5.1).

**TABLE 2:** Association between type 1 diabetes mellitus and the risk for preeclampsia stratified by alternative measures of poor glycaemic control and diabetes severity.

Measure	Mothers total, *n*	Preeclampsia, *n* (%)	Unadjusted risk ratio (95% CI)	Adjusted risk ratio^a^ (95% CI)
No diabetes (common reference in all analyses)	1 684 872	49 024 (2.9)	Reference	Reference
Hospital admission for acidosis^c^ in the year before conception				
Yes	47	10 (21.3)	6.9 (3.9–12.34)	5.4 (3.2–9.0)
No	4382	736 (16.8)	5.7 (5.3–6.1)	4.7 (4.4–5.0)
Hospital admission for T1DM in the year before conception				
Yes	218	37 (17.0)	5.8 (4.3–7.7)	4.5 (3.4–6.0)
No	4211	709 (16.8)	5.7 (5.3–6.1)	4.7 (4.4–5.1)
Long‐term glycaemic control (average of the last three HbA1c values, mmol/mol)^b^				
< 48	584	51 (8.7)	3.0 (2.3–3.9)	2.9 (2.2–3.7)
48–61	1941	287 (14.8)	5.0 (4.5–5.6)	4.3 (3.8–4.7)
62–75	1058	218 (20.6)	7.0 (6.2–7.9)	5.2 (4.58–5.85)
≥ 76	381	113 (29.7)	9.9 (8.5–11.6)	7.3 (6.26–8.62)
Albuminuria (renal disease)				
Normoalbuminuria	3641	552 (15.2)	5.2 (4.8–5.6)	4.2 (3.9–4.6)
Microalbuminuria	663	160 (24.1)	8.1 (7.0–9.3)	6.8 (5.9–7.9)
Macroalbuminuria	125	34 (27.2)	9.0 (6.6–12.2)	7.1 (5.3–9.5)
Estimated glomerular filtration rate (mL/min)				
> 120	820	162 (19.8)	6.6 (5.8–7.6)	5.2 (4.6–6.0)
60–120	3389	539 (15.9)	5.4 (5.0–5.9)	4.5 (4.1–4.9)
< 60	73	18 (24.7)	8.3 (5.5–12.5)	7.2 (4.8–10.9)

Abbreviations: HbA1c, haemoglobin A1c (glycated haemoglobin); T1DM, Type 1 diabetes mellitus.

^a^ Adjusted for year of birth, maternal age, country of birth, living with a partner, education level, parity, body mass index, assisted reproductive technology, smoking status, other diabetes‐related autoimmune diseases.

^b^ Most HbA1c values were retrieved earlier than 90 days before conception.

^c^ Acidosis defined as having a hospital admission with a primary diagnosis (in the National Patient Register) of ICD‐10: E10.0A, E10.0B, E10.0D, E10.0X, E10.1A, E10.1B, E10.1D, E10.1X, E11.0A, E11.0B, E11.0D, E11.0X, E11.1A, E11.1B, E11.1D, E11.1X.

The aRR of preeclampsia tended to be higher among women with T1DM and macroalbuminuria (27.2%; aRR 7.1 (5.3–9.5)) and a reduced glomerular filtration rate (< 60 mL/min) (24.7%; aRR 7.2, 4.8–10.9) (Table 2). Regarding long‐term glycaemic control (mean of the last three HbA1c measurements), the incidence and risk of preeclampsia increased with higher mean HbA1c levels; rising from 8.7% at HbA1c < 48 mmol/mol (aRR 2.9, 2.2–3.7) to 29.7% at HbA1c ≥ 76 mmol/mol (aRR 7.3, 6.3–8.6).

The Kaplan–Meier failure curve for time to preeclampsia, shown from gestational Week 20 to 1 week postpartum (Figure S3a), indicates that the cumulative probability of developing preeclampsia began to diverge between women with and without T1DM around 28 weeks of gestation. Among women with T1DM, the cumulative risk of preeclampsia increased more rapidly with higher HbA1c levels (Figure S3b). Stratified by parity, similar trends were observed but with a higher cumulative risk for nulliparous women (Figure S4a) than for parous women (Figure S4b).

## 4 Discussion

### 4.1 Main Findings

In this population‐based study of 1 689 301 singleton births in Sweden between 2003 and 2019, the overall adjusted risk ratio of preeclampsia was 4.7 times higher among the 4429 pregnancies in women with T1DM, compared to women without diabetes. While T1DM was a strong risk factor for developing preeclampsia, this risk also increased in a dose–response pattern with poorer glycaemic control, as measured by increased HbA1c levels around the start of the pregnancy (conception). Compared to women without diabetes, the highest risk of developing preeclampsia was seen among women with T1DM and higher HbA1c levels (≥ 76 mmol/mol), who overall had more than a six‐fold increased risk. The association between HbA1c levels above the target range (≥ 48 mmol/mol according to several national guidelines) [44, 45] and preeclampsia was strongest for early (delivery < 34 gestational weeks) and late (delivery 34–36 gestational weeks) preterm preeclampsia. While we have shown that poor glycaemic control is associated with an increased risk of preeclampsia, we also found a three‐fold risk of developing preeclampsia in women with T1DM and HbA1c levels within the target range. This simultaneously highlights the inherent increased risk of preeclampsia in women with T1DM, as well as the importance of optimising peri‐conceptional glycaemic control to provide the lowest risk possible.

### 4.2 Interpretation

The overall incidence of preeclampsia in women with T1DM in this study (16.8%) is comparable to a 2018 systematic review (based on 11 studies and over 10 000 pregnancies), where the risk was 17% (ranging from 9.3% to 33.6%) [26]. An intervention study aiming to improve glycaemic control in women with T1DM found poor glycaemic control was associated with a higher preeclampsia risk [27]. Another study (290 pregnancies in 178 women) identified elevated HbA1c levels at 24 weeks' gestation as the strongest predictor of preeclampsia in women with T1DM [23]. In this study, HbA1c levels within 3 months before and after conception were used to assess glycaemic control; however, differences pre‐ or post‐conception were not examined. While smaller studies have reported similar findings, with elevated first‐trimester HbA1c levels predicting preeclampsia among women with T1DM [46], no study to date has had a sufficiently large sample to evaluate preeclampsia risk across HbA1c levels while adjusting for important confounders [26]. Our large population‐based study, with prospectively collected HbA1c measurements around conception and detailed pregnancy and birth outcomes, holds significant value. The robust sample size provided high statistical power, while the unique data enabled an in‐depth examination of glycaemic control before and around conception.

Women with T1DM who did not achieve optimal glycaemic control around conception faced a higher risk of preeclampsia, with a positive dose–response between HbA1c levels and risk, especially for preterm preeclampsia. The risk of preeclampsia was, however, only marginally influenced by other measures of T1DM severity, such as ketoacidosis or admission to hospital for T1DM in the 12 months before conception. For women with T1DM, maintaining optimal glycaemic control within target range around conception offers the lowest risk of preeclampsia. While we saw a trend of better glycaemic control among the women in our sample over time, encouraging women with T1DM of reproductive age to maintain good glycaemic control should continue to be prioritised. While pre‐conception pregnancy care programmes for women with T1DM are effective, both clinically and from a cost perspective [47], greater effort is needed to engage women and support increased uptake of these health‐promoting services.

## 5 Strengths and Limitations

This study's strengths include using routinely and prospectively collected nationwide data, linked across multiple high‐quality, validated registers [29, 30, 48], including the National Diabetes Register, the National Patient Register and the Medical Birth Register. The large sample size enabled accurate identification of women with T1DM (using diagnoses in the National Diabetes Register [49]) and assessment of HbA1c levels, minimising exposure misclassification. The extensive population provided high statistical power to examine the risk of preeclampsia by gestation and HbA1c levels. Well‐defined pregnancy and maternal characteristics facilitated adjustment for potential confounders. The national, population‐based design further enhanced the generalisability of our findings.

A key limitation is that the NDR typically records HbA1c levels once annually, typically during yearly clinical visits. To assess glycaemic control around conception and placentation, we included women with HbA1c measured around conception. This excluded some women with T1DM, possibly introducing selection bias. Diagnosing preeclampsia in a woman with T1DM who already has albuminuria can be complex due to overlapping features, and that increasing proteinuria after gestational week 20 in women with proteinuria, combined with hypertension, is part of the diagnostic criteria for preeclampsia. However, in our sensitivity analysis excluding women with albuminuria in the last 12 months before conception, neither the direction nor interpretation of our main findings were changed, despite slightly lower estimates. This was also the case in our sensitivity analysis adjusting for aspirin use during pregnancy (from 2006 onwards, due to data availability in the Prescribed Drug Register), since low‐dose aspirin use from gestational week 12 can reduce the risk of developing preterm preeclampsia [50].

Another limitation is the lack of data from continuous glucose monitoring (CGM) as a measure of glycaemic control during pregnancy. While CGM is now widely used, we did not have access to such data in the present study. Although HbA1c continues to serve as a useful and clinically used summary measure, CGM should be considered a superior tool for optimising and individualising glycaemic management in pregnancy. Estimated glomerular filtration rate (eGFR) is generally accepted as the best overall indicator of renal function. However, pregnancy increases kidney filtration, lowering serum creatinine and raising eGFR values, which also differ by trimester. Therefore, eGFR should be interpreted with caution, alongside other clinical and biochemical markers. Differences in maternal and behavioural characteristics between women with and without T1DM, and within women with T1DM, mean that there could also be residual confounding. Additionally, our findings may not be generalisable to populations outside Sweden.

Having T1DM was a strong risk factor for developing preeclampsia, with glycaemic control having a dose‐dependent association. Women with T1DM who did not achieve optimal glycaemic control around conception faced a higher risk of preeclampsia, with a positive dose–response between HbA1c levels and risk, especially for preterm preeclampsia. While even those with guideline‐recommended HbA1c levels had a higher risk of preeclampsia compared to women without diabetes, maintaining optimal glycaemic control within target range around conception offers the lowest risk of preeclampsia among women with T1DM.

## Author Contributions

N.R., N.H., B.P., J.S., M.N., S.G., A.S., J.F.L., O.S.: conceptualisation; J.S.: data curation and formal analysis; N.H., N.R.: writing (original draft); N.H., N.R., B.P., J.S., M.N., S.G., A.S., J.F.L., O.S.: writing (review and editing). All authors have read and approved the final manuscript and accept responsibility for the paper as published.

## Ethics Statement

This study was approved by the Swedish Ethical Review Authority (approval 2024‐02989‐01) and did not require consent to use the MBR. All women registered in the National Diabetes Register provided informed consent.

## Conflicts of Interest

The authors declare no conflicts of interest.

## Supporting information


**Figure S1:** Risk ratios and risk differences of pre‐eclampsia, overall and stratified by timing of pre‐eclampsia onset, by T1DM status and levels of HbA1c in *nulliparous* women.
**Figure S2:** Risk ratios and risk differences of pre‐eclampsia, overall and stratified by timing of pre‐eclampsia onset, by T1DM status and levels of HbA1c in *parous* women.
**Figure S3:** (a) Kaplan–Meier failure curves of time to preeclampsia from gestational week 20 until 1 week postpartum, singleton pregnancies in Sweden between 2003–2019. (b) Kaplan–Meier failure curves of time to preeclampsia from gestational week 20 until 1 week postpartum by HbA1c (glycated haemoglobin) levels, singleton pregnancies in Sweden between 2003 and 2019.
**Figure S4:** (a) Kaplan–Meier failure curves of time to preeclampsia from gestational week 20 until 1 week postpartum by HbA1c (glycated haemoglobin) levels, in singleton *nulliparous* pregnancies in Sweden between 2003 and 2019. (b) Kaplan–Meier failure curves of time to preeclampsia from gestational week 20 until 1 week postpartum by HbA1c (glycated haemoglobin) levels, in singleton *parous* pregnancies in Sweden between 2003 and 2019.
**Figure S5:** Risk ratios and risk differences of preeclampsia by Type 1 diabetes mellitus (T1DM) status and levels of HbA1c, main and sensitivity analyses (births from 2006; births from 2006 adjusted for aspirin use; and excluding women with albuminuria).
**Table S1:** Criteria and definitions of the exposure (diabetes and non‐diabetes) and main outcome.
**Table S2:** Definition of Subgroups for the analyses using alternative measures of poor glycaemic control and diabetes severity.
**Table S3:** Covariates included in the multivariable model, including definition/categories, data sources and extent of missing values.
**Table S4:** Maternal characteristics stratified by Type 1 diabetes mellitus (T1DM) status and levels of glycated haemoglobin (HbA1c) in non‐imputed data (*N*=1,689,301).
**Table S5:** Maternal characteristics of women with Type 1 diabetes mellitus (T1DM), stratified by whether or not they had at least one peri‐conceptional HbA1c measurement (within 3 months before and after conception).

## Data Availability

The data that support the findings of this study are available from the corresponding author upon reasonable request.
