# Supplementary material for: Glycaemic Control in Women With Type 1 Diabetes and Preeclampsia Risk: A Nationwide Cohort Study
Source: BJOG. 2025 Aug 26;133(1):95–105. doi: 10.1111/1471-0528.18339 (PMC12676193; doi:10.1111/1471-0528.18339)
Supplement: Supplementary file 1 — Figure S1: Risk ratios and risk differences of pre‐eclampsia, overall and stratified by timing of pre‐eclampsia onset, by T1DM status and levels of HbA1c in nulliparous women. Figure S2: Risk ratios and risk differences of pre‐eclampsia, overall and stratified by timing of pre‐eclampsia onset, by T1DM status and levels of HbA1c in parous women. Figure S3: (a) Kaplan–Meier failure curves of time to preeclampsia from gestational week 20 until 1 week postpartum, singleton pregnancies in Sweden between 2003–2019. (b) Kaplan–Meier failure curves of time to preeclampsia from gestational week 20 until 1 week postpartum by HbA1c (glycated haemoglobin) levels, singleton pregnancies in Sweden between 2003 and 2019. Figure S4: (a) Kaplan–Meier failure curves of time to preeclampsia from gestational week 20 until 1 week postpartum by HbA1c (glycated haemoglobin) levels, in singleton nulliparous pregnancies in Sweden between 2003 and 2019. (b) Kaplan–Meier failure curves of time to preeclampsia from gestational week 20 until 1 week postpartum by HbA1c (glycated haemoglobin) levels, in singleton parous pregnancies in Sweden between 2003 and 2019. Figure S5: Risk ratios and risk differences of preeclampsia by Type 1 diabetes mellitus (T1DM) status and levels of HbA1c, main and sensitivity analyses (births from 2006; births from 2006 adjusted for aspirin use; and excluding women with albuminuria). Table S1: Criteria and definitions of the exposure (diabetes and non‐diabetes) and main outcome. Table S2: Definition of Subgroups for the analyses using alternative measures of poor glycaemic control and diabetes severity. Table S3: Covariates included in the multivariable model, including definition/categories, data sources and extent of missing values. Table S4: Maternal characteristics stratified by Type 1 diabetes mellitus (T1DM) status and levels of glycated haemoglobin (HbA1c) in non‐imputed data (N=1,689,301). Table S5: Maternal characteristics of women with Type 1 diabetes mellit [file BJO-133-95-s001.docx]

Supplementary Tables and Figures

**eTable 1** Criteria and definitions of the exposure (diabetes and non-diabetes) and main outcome

|  | **Data source** | **Code** | **Description** |
| --- | --- | --- | --- |
| **Diabetes** | National Patient register | ICD-10: E10-E14, O24 | Type 1, type 2, other diabetes, gestational diabetes |
|  | Medical Birth Register | ICD-10: O24 | Diabetes during pregnancy (including type 1, type 2, other diabetes, gestational diabetes) |
|  | National Diabetes Register | 1, 2, 3 | Type 1, type 2 and other diabetes |
|  | Prescribed drug register | ATC-codes: A10A, A10B | Type 1, type 2 and other diabetes |
| **Preeclampsia** | National Patient register | ICD-10: O11, O14-O15 | Preeclampsia and eclampsia |
|  | Medical Birth Register | ICD-10: O11, O14-O15 | Preeclampsia and eclampsia |
| **Chronic hypertension** | National Patient register | ICD-10: O10, I10-I15 | Hypertension before conception |
|  | Medical Birth Register | ICD-10: O10 | Hypertension before conception |
|  | Prescribed Drug Register | ATC-codes: C02, C03, C07, C08, C09 | Hypertension before conception |
| **SGA** | Medical Birth Register | ICD-10: O36.5  Birth weight | Intrauterine growth restriction  Small for gestational age (SGA), defined as birth weight < 2 standard deviations below the sex-specific mean for gestational age |

**eTable 2** Definition of Subgroups for the analyses using alternative measures of poor glycaemic control and diabetes severity

| **Subgroup** | **Categories (reference category in all analyses is pregnancies without diabetes)** | **Definition** |
| --- | --- | --- |
| **Hospital admission due to acidosis**  **(in the year before conception)** | yes (≥1)/no | Hospital admission with a primary diagnosis of ICD-10 E10.0A, E10.0B, E10.0D, E10.0X, E10.1A, E10.1B, E10.1D, E10.1X, E11.0A, E11.0B, E11.0D, E11.0X, E11.1A, E11.1B, E11.1D, E11.1X.  Data from the National Patient Register |
| **Hospital admission due to diabetes**  **(in the year before conception)** | yes (≥1)/no | Hospital admission with ICD-10 E10, E11, E14 as primary diagnosis.  Data from the National Patient Register |
| **Long-term glycaemic control** | Glycated haemoglobin (HbA1c; mmol/mol) <48, 48 to 61, 62 to 75, ≥76 | Mean of last 3 values, restricted to individuals with at least 3 values, each registered at least 6 months apart. Since we required at least 6 months between HbA1c values, these were not restricted to measures during pregnancy.  Data from National Diabetes Register. |
| **Albuminuria ^a^** | Normoalbuminuria; Microalbuminuria*; Macroalbuminuria# | According to last registered value.  Data from the National Diabetes Register |
| **Estimated glomerular filtration rate (eGRF) (ml/min/1.73m2)** | >120; 60-120; <60 | Using last registered creatinine value in the National Diabetes Register. Calculated by the Modification of Diet in Renal Disease equation. |
| **Hypoglycaemia** | yes (≥1)/no | ICD-10: E15.9, E10.0C, E10.6A, E11.0C, E11.6A, E16.0-E16.2  Any type of diagnosis, any type of hospital contact |
| **Ketoacidosis** | yes (≥1)/no | ICD-10: E10.0A, E10.1  Any type of diagnosis, any type of hospital contact |

**^a^Albuminuria defined using ICD-10 code R80 in the patient register (any type or any hospital contact)**

* Microalbuminuria was defined as having ≥2 positive results from 3 urine samples obtained within 1 year, with positivity defined as an albumin-to-creatinine ratio of 3–30 mg/mmol (roughly 30–300 mg/g) or urinary albumin clearance of 20–200 μg/min (20–300 mg/L).

# Macroalbuminuria was defined as having an albumin-to-creatinine ratio of ˃30 mg/mmol (roughly ≥300 mg/g) or urinary albumin clearance of ˃200 μg/min (>300 mg/L).

**eTable 3** Covariates included in the multivariable model, including definition/categories, data sources, and extent of missing values

| **Characteristic** | **Definition/categories** |
| --- | --- |
| Maternal age at conception | Categorical (Descriptive), continuous (modelling) |
| Country of birth | Nordic/Other |
| Living with a partner | Yes/No |
| Year of birth | 2003-2006, 2007-2010, 2011-2014 |
| Highest achieved education (years)# | ≤9, 10-12, ≥13 |
| Parity | 0, 1, ≥2 |
| Body mass index early pregnancy* | <18.5, 18.5 to <25, 25 to <30, 30 to <35, ≥35 |
| ART* | Yes/No |
| Smoking in early pregnancy* | Yes/No |
| Hypertension **§** | ICD-10: ICD-10: O10, I10-I15 |
| Other diabetes-related autoimmune diseases **§** (autoimmune thyroiditis, Grave’s disease, celiac disease, Addison’s disease, autoimmune polyglandular syndrome, rheumatoid arthritis and juvenile arthritis, systemic lupus erythematosus, pernicious anaemia, primary biliary cirrhosis, ulcerative colitis, Wegener’s granulomatosis, psoriasis) | ICD-10: D510, E050, E063, E271, E272, E310, K51, K743, K900, L40, M05, M06, M08, M313, M32 Any type of diagnosis, any type of hospital contact |
| Aspirin use | ATC: B01AC06  Data from the Prescribed Drug Register |

Data sources: * Medical Birth Register. # Education Register. § National Patient Register.

**eTable 4.** Maternal characteristics stratified by Type 1 diabetes mellitus (T1DM) status and levels of glycated haemoglobin (HbA1c) in non-imputed data (N=1,689,301)

| **Characteristics** | **Mothers without diabetes**  (n=1,684,872) | **Mothers with T1DM, stratified by levels of glycated hemoglobin (HbA1c)** | | | | |
| --- | --- | --- | --- | --- | --- | --- |
|  |  | **Total**  (n=4,429) | **HbA1c <48 mmol/mol**  (n=1,118) | **HbA1c 48-61 mmol/mol**  (n=2,037) | **HbA1c 62-75 mmol/mol**  (n=892) | **HbA1c ≥76 mmol/mol**  (n=382) |
| **Age, years, mean (SD)** | 30.2 (5.0) | 29.9 (4.8) | 30.1 (4.4) | 30.3 (4.7) | 29.6 (5.1) | 27.8 (5.3) |
| 18-24.9 | 272,719 (16.2) | 697 (15.7) | 126 (11.3) | 262 (12.9) | 181 (20.3) | 128 (33.5) |
| 25-29.9 | 554,415 (32.9) | 1,620 (36.6) | 431 (38.6) | 747 (36.7) | 310 (34.8) | 132 (34.6) |
| 30-34.9 | 561,320 (33.3) | 1,409 (31.8) | 396 (35.4) | 678 (33.3) | 256 (28.7) | 79 (20.7) |
| 35-39.9 | 251,954 (15.0) | 603 (13.6) | 143 (12.8) | 302 (14.8) | 122 (13.7) | 36 (9.4) |
| ≥40 | 44,464 (2.6) | 100 (2.3) | 22 (2.0) | 48 (2.4) | 23 (2.6) | 7 (1.8) |
| **Country of birth** |  |  |  |  |  |  |
| Nordic | 1,339,080 (79.5) | 4,167 (94.1) | 1,051 (94.0) | 1,936 (95.0) | 832 (93.3) | 348 (91.1) |
| Other | 343,551 (20.4) | 258 (5.8) | 66 (5.9) | 100 (4.9) | 58 (6.5) | 34 (8.9) |
| Missing | 2,241 (0.1) | 4 (0.1) | 1 (0.1) | 1 (0.0) | 2 (0.2) | 0 |
| **Living with a partner** |  |  |  |  |  |  |
| Yes | 1,507,885 (89.5) | 4,003 (90.4) | 1,037 (92.8) | 1,872 (91.9) | 786 (88.1) | 308 (80.6) |
| No | 94,466 (5.6) | 236 (5.3) | 39 (3.5) | 73 (3.6) | 72 (8.1) | 52 (13.6) |
| Missing | 82,521 (4.9) | 190 (4.3) | 42 (3.8) | 92 (4.5) | 34 (3.8) | 22 (5.8) |
| **Year of birth** |  |  |  |  |  |  |
| 2003-2009 | 658,494 (39.1) | 1,116 (25.2) | 262 (23.4) | 500 (24.5) | 254 (28.5) | 100 (26.2) |
| 2010-2014 | 510,144 (30.3) | 1,378 (31.1) | 293 (26.2) | 624 (30.6) | 306 (34.3) | 155 (40.6) |
| 2015-2019 | 516,234 (30.6) | 1,935 (43.7) | 563 (50.4) | 913 (44.8) | 332 (37.2) | 127 (33.2) |
| **Level of education, years** |  |  |  |  |  |  |
| ≤9 | 173,062 (10.3) | 358 (8.1) | 50 (4.5) | 127 (6.2) | 97 (10.9) | 84 (22.0) |
| 10-12 | 646,359 (38.4) | 1,810 (40.9) | 372 (33.3) | 791 (38.8) | 436 (48.9) | 211 (55.2) |
| ≥13 | 854,656 (50.7) | 2,252 (50.8) | 694 (62.1) | 1,115 (54.7) | 357 (40.0) | 86 (22.5) |
| Missing | 10,795 (0.6) | 9 (0.2) | 2 (0.2) | 4 (0.2) | 2 (0.2) | 1 (0.3) |
| **Parity** |  |  |  |  |  |  |
| 0 | 731,826 (43.4) | 2,157 (48.7) | 567 (50.7) | 1,008 (49.5) | 406 (45.5) | 176 (46.1) |
| 1 | 629,382 (37.4) | 1,629 (36.8) | 432 (38.6) | 746 (36.6) | 318 (35.7) | 133 (34.8) |
| ≥2 | 323,664 (19.2) | 643 (14.5) | 119 (10.6) | 283 (13.9) | 168 (18.8) | 73 (19.1) |
| **Body mass index in early pregnancy, kg/m^2^** |  |  |  |  |  |  |
| <18.5 | 36,894 (2.2) | 21 (0.5) | 4 (0.4) | 8 (0.4) | 6 (0.7) | 3 (0.8) |
| 18.5-<25 | 935,184 (55.5) | 1,941 (43.8) | 557 (49.8) | 877 (43.1) | 359 (40.2) | 148 (38.7) |
| 25-<30 | 396,300 (23.5) | 1,511 (34.1) | 374 (33.5) | 705 (34.6) | 304 (34.1) | 128 (33.5) |
| 30-<35 | 136,812 (8.1) | 536 (12.1) | 110 (9.8) | 256 (12.6) | 113 (12.7) | 57 (14.9) |
| ≥35 | 56,167 (3.3) | 180 (4.1) | 30 (2.7) | 78 (3.8) | 50 (5.6) | 22 (5.8) |
| Missing | 123,515 (7.3) | 240 (5.4) | 43 (3.8) | 113 (5.5) | 60 (6.7) | 24 (6.3) |
| **Assisted reproductive technology** | 54,393 (3.2) | 160 (3.6) | 57 (5.1) | 75 (3.7) | 25 (2.8) | 3 (0.8) |
| **Smoking in early pregnancy** |  |  |  |  |  |  |
| Non-smoker | 1,496,701 (88.8) | 3,929 (88.7) | 1,032 (92.3) | 1,852 (90.9) | 768 (86.1) | 277 (72.5) |
| 1-9 cigarettes/day | 75,977 (4.5) | 188 (4.2) | 23 (2.1) | 52 (2.6) | 60 (6.7) | 53 (13.9) |
| ≥10 cigarettes/day | 22,546 (1.3) | 58 (1.3) | 7 (0.6) | 14 (0.7) | 17 (1.9) | 20 (5.2) |
| Missing | 89,648 (5.3) | 254 (5.7) | 56 (5.0) | 119 (5.8) | 47 (5.3) | 32 (8.4) |
| **Pre-pregnancy hypertension** | 66,688 (4.0) | 812 (18.3) | 145 (13.0) | 378 (18.6) | 192 (21.5) | 97 (25.4) |
| **Other diabetes-related autoimmune diseases** | 35,464 (2.1) | 595 (13.4) | 144 (12.9) | 256 (12.6) | 140 (15.7) | 55 (14.4) |
| **Median duration T1DM (IQR), years** | - | 16 (9-22) | 14 (5-20) | 16 (10-22) | 16 (11-22) | 15 (10-20) |
| **Median HbA1c level (IQR),mmol/mol** | - | 55 (47-64) | 43 (39-45) | 54 (51-58) | 67 (64-70) | 83 (79-91) |

T1DM = Type 1 diabetes mellitus; HbA1c = haemoglobin A1c (glycated haemoglobin); IQR = interquartile range; SD= Standard deviation.

^a^1,003,194 unique women without diabetes ^b^ 3,428 unique women with diabetes.

**eTable 5.** Maternal characteristics of women with Type 1 diabetes mellitus (T1DM), stratified by whether or not they had at least one periconceptional HbA1c measurement (within 3 months before and after conception)

| **Characteristic** | **Mother with Type 1 Diabetes and at least one HbA1c value**  (n=4,429) | **Mother with Type 1 Diabetes and no HbA1c value**  (n=971) |
| --- | --- | --- |
| **Age, years, mean (SD)** | 29.9 (4.8) | 30.4 (4.9) |
| 18-24.9 | 697 (15.7) | 140 (14.4) |
| 25-29 | 1,620 (36.6) | 333 (34.3) |
| 30-34 | 1,409 (31.8) | 325 (33.5) |
| 35-39 | 603 (13.6) | 149 (15.3) |
| ≥40 | 100 (2.3) | 24 (2.5) |
| **Country of birth** |  |  |
| Nordic | 4,167 (94.1) | 883 (90.9) |
| Other | 258 (5.8) | 86 (8.9) |
| Missing | 4 (0.1) | 2 (0.2) |
| **Living with partner** |  |  |
| Yes | 4,003 (90.4) | 848 (87.3) |
| No | 236 (5.3) | 70 (7.2) |
| Missing | 190 (4.3) | 53 (5.5) |
| **Year of birth** |  |  |
| 2003-2009 | 1,116 (25.2) | 195 (20.1) |
| 2010-2014 | 1,378 (31.1) | 314 (32.3) |
| 2015-2019 | 1,935 (43.7) | 462 (47.6) |
| **Level of education, years** |  |  |
| ≤9 | 358 (8.1) | 96 (9.9) |
| 10-12 | 1,810 (40.9) | 355 (36.6) |
| ≥13 | 2,252 (50.8) | 515 (53.0) |
| Missing | 9 (0.2) | 5 (0.5) |
| **Parity** |  |  |
| 0 | 2,157 (48.7) | 425 (43.8) |
| 1 | 1,629 (36.8) | 369 (38.0) |
| ≥2 | 643 (14.5) | 177 (18.2) |
| **Body mass index in early pregnancy, kg/m^2^** |  |  |
| <18.5 | 21 (0.5) | 8 (0.8) |
| 18.5-24 | 1,941 (43.8) | 424 (43.7) |
| 25-29 | 1,511 (34.1) | 302 (31.1) |
| 30-34 | 536 (12.1) | 132 (13.6) |
| ≥35 | 180 (4.1) | 39 (4.0) |
| Missing | 240 (5.4) | 66 (6.8) |
| **Assisted reproductive technology** | 160 (3.6) | 42 (4.3) |
| **Smoking in early pregnancy** |  |  |
| Non-smoker | 3,929 (88.7) | 828 (85.3) |
| 1-9 cigarettes/day | 188 (4.2) | 55 (5.7) |
| ≥10 cigarettes/day | 58 (1.3) | 24 (2.5) |
| Missing | 254 (5.7) | 64 (6.6) |
| **Pre-pregnancy hypertension** | 812 (18.3) | 202 (20.8) |
| **Other autoimmune disease** | 595 (13.4) | 128 (13.2) |
| **Median duration T1DM (IQR), years** | 16 (9-22) | 17 (11-23) |
| **Median HbA1c level (IQR), mmol/mol** | 55 (47-64) | - |

T1DM = Type 1 diabetes mellitus; HbA1c = haemoglobin A1c (glycated haemoglobin); IQR = interquartile range; SD= Standard deviation.


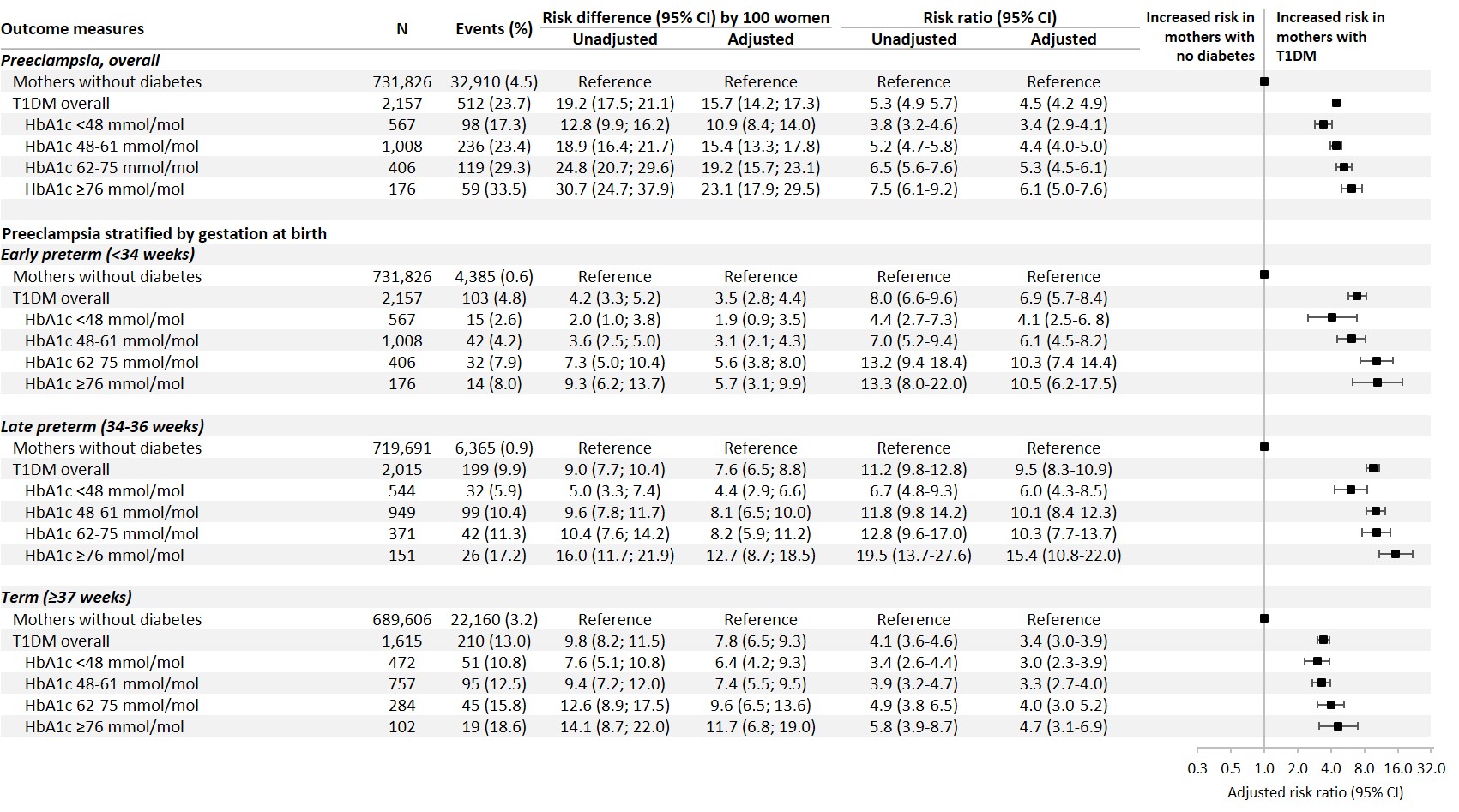


**eFigure 1.** Risk ratios and risk differences of preeclampsia, overall and stratified by timing of preeclampsia onset,
by T1DM status and levels of HbA1c in nulliparous women.

**
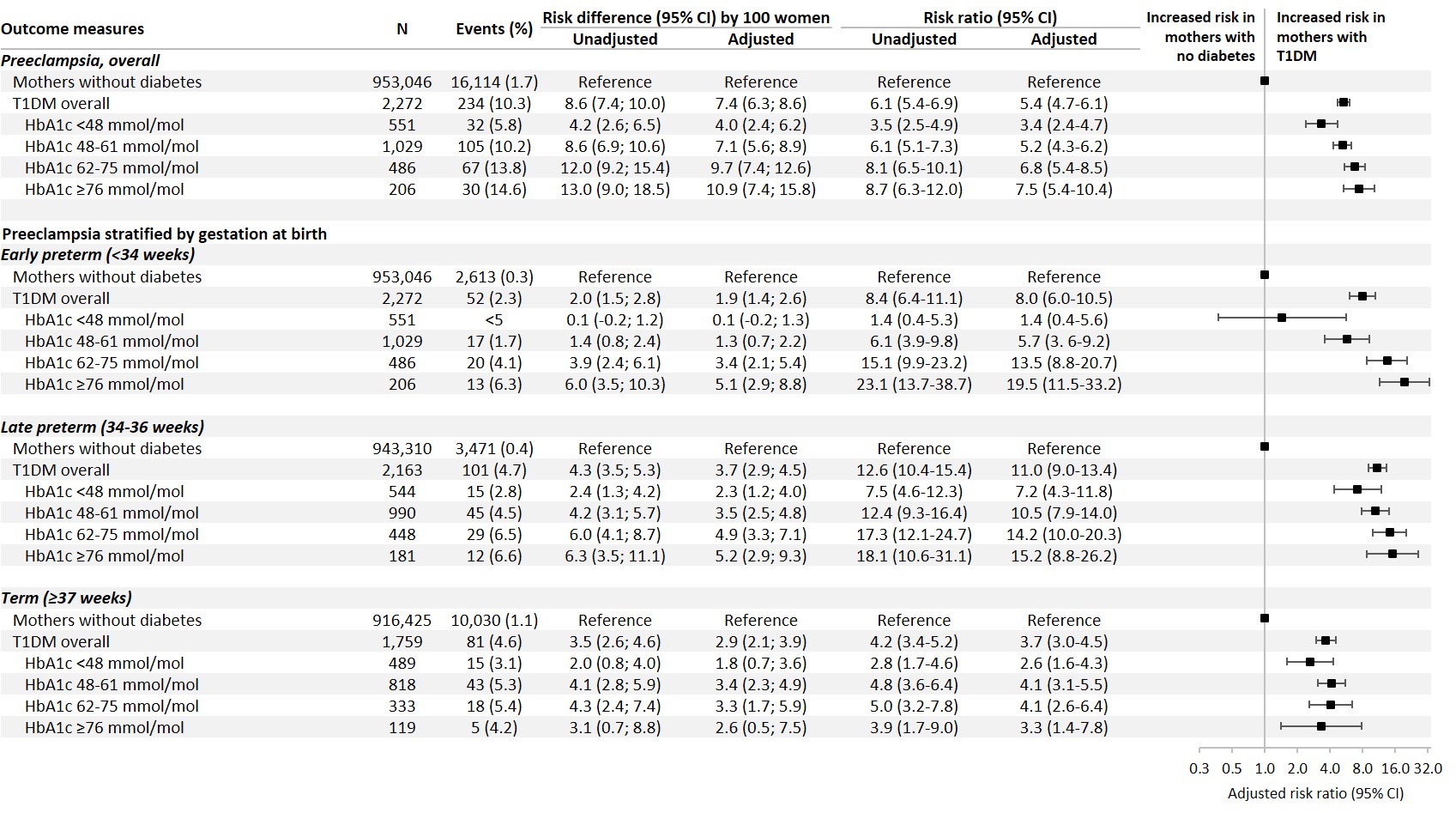
eFigure 2**. Risk ratios and risk differences of preeclampsia, overall and stratified by timing of preeclampsia onset,
by T1DM status and levels of HbA1c in parous women.


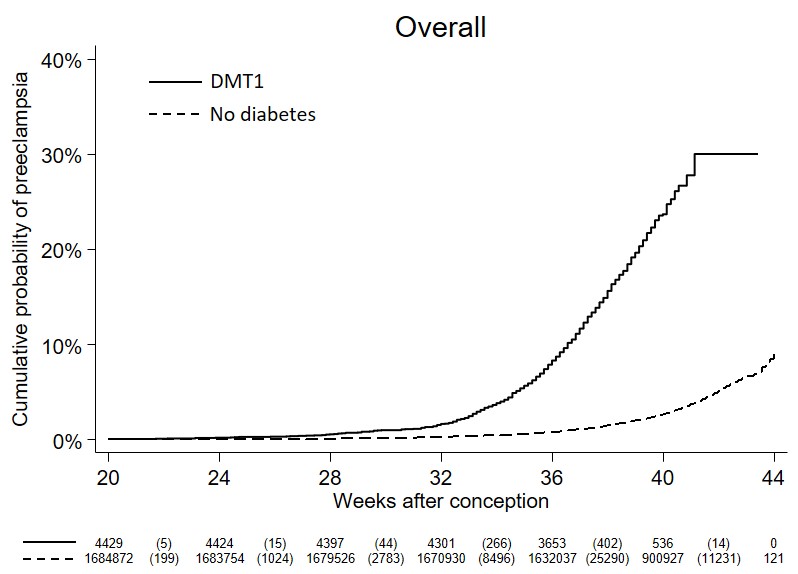


**eFigure 3a.** Kaplan-Meier failure curves of time to preeclampsia from gestational week 20 until 1 week postpartum, singleton pregnancies in Sweden between 2003-2019.

**
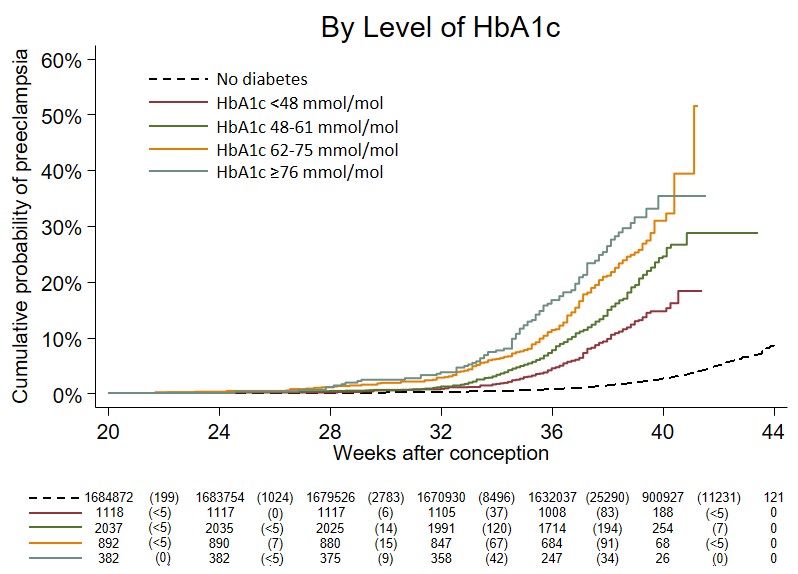
**

**eFigure 3b.** Kaplan-Meier failure curves of time to preeclampsia from gestational week 20 until 1 week postpartum by HbA1c (glycated haemoglobin) levels, singleton pregnancies in Sweden between 2003-2019.

**
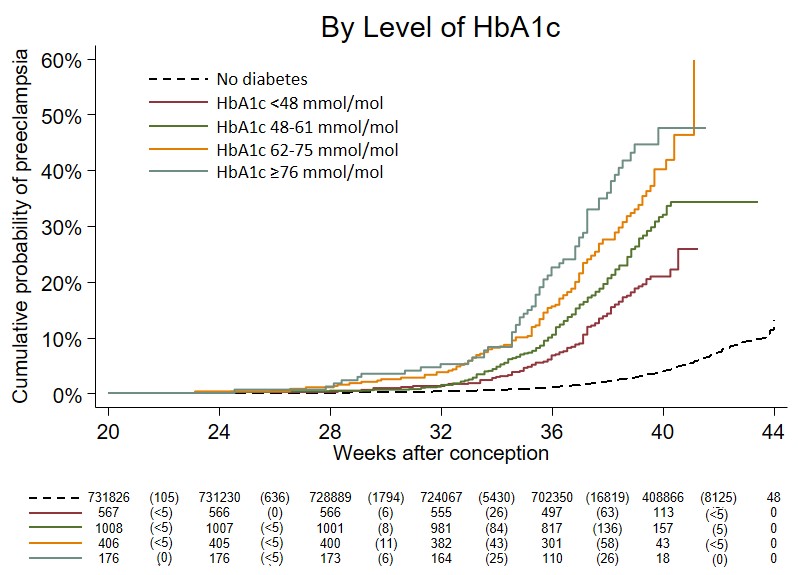
**

**eFigure 4a.** Kaplan-Meier failure curves of time to preeclampsia from gestational week 20 until 1 week postpartum by HbA1c (glycated haemoglobin) levels, in singleton nulliparous pregnancies in Sweden between 2003-2019.

**
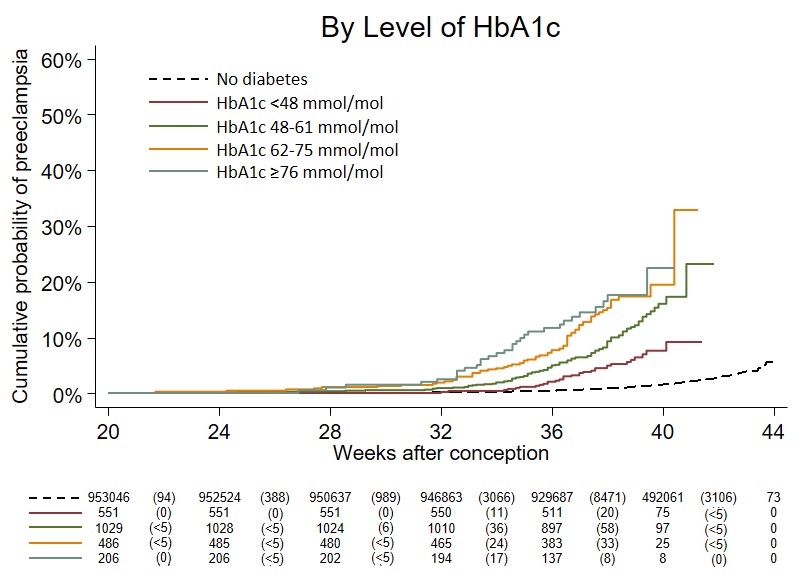
**

**eFigure 4b.** Kaplan-Meier failure curves of time to preeclampsia from gestational week 20 until 1 week postpartum by HbA1c (glycated haemoglobin) levels, in singleton parous pregnancies in Sweden between 2003-2019.


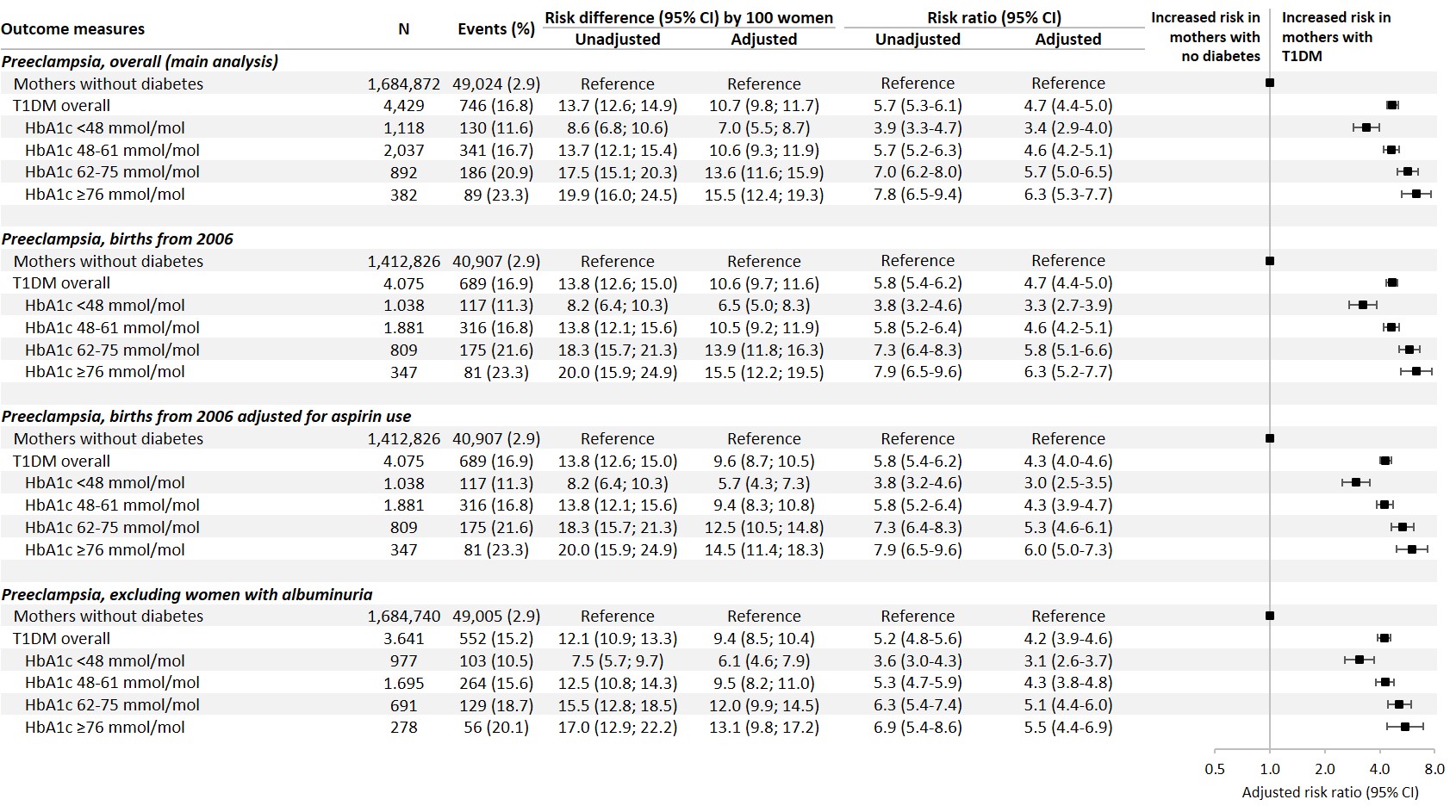


**eFigure 5**. Risk ratios and risk differences of preeclampsia by Type 1 diabetes mellitus (T1DM) status and levels of HbA1c, main and sensitivity analyses (births from 2006; births from 2006 adjusted for aspirin use; and excluding women with albuminuria within 12 months before conception).

Albuminuria defined as Normoalbuminuria, Mircoalbuminuria, or Macroalbuminuria, based on the last value in the National Diabetes Register - Microalbuminuria (≥2 positive results from 3 urine samples obtained within 1 year, with positivity defined as an albumin-to-creatinine ratio of 3–30 mg/mmol (roughly 30–300 mg/g) or urinary albumin clearance of 20–200 μg/min (20–300 mg/L)); Macroalbuminuria (having an albumin-to-creatinine ratio of ˃30 mg/mmol (roughly ≥300 mg/g) or urinary albumin clearance of ˃200 μg/min (>300 mg/L)).
